# An Entropy Metric for Regular Grammar Classification and Learning with Recurrent Neural Networks

**DOI:** 10.3390/e23010127

**Published:** 2021-01-19

**Authors:** Kaixuan Zhang, Qinglong Wang, C. Lee Giles

**Affiliations:** 1 Information Sciences and Technology, Pennsylvania State University, University Park, PA 16802, USA; kuz22@psu.edu; 2 Alibaba Group, Building A2, Lane 55 Chuan He Road Zhangjiang, Pudong New District, Shanghai 200135, China; xifu.wql@alibaba-inc.com

**Keywords:** entropy, regular grammar classification, complexity analysis, recurrent neural network

## Abstract

Recently, there has been a resurgence of formal language theory in deep learning research. However, most research focused on the more practical problems of attempting to represent symbolic knowledge by machine learning. In contrast, there has been limited research on exploring the fundamental connection between them. To obtain a better understanding of the internal structures of regular grammars and their corresponding complexity, we focus on categorizing regular grammars by using both theoretical analysis and empirical evidence. Specifically, motivated by the concentric ring representation, we relaxed the original order information and introduced an entropy metric for describing the complexity of different regular grammars. Based on the entropy metric, we categorized regular grammars into three disjoint subclasses: the polynomial, exponential and proportional classes. In addition, several classification theorems are provided for different representations of regular grammars. Our analysis was validated by examining the process of learning grammars with multiple recurrent neural networks. Our results show that as expected more complex grammars are generally more difficult to learn.

## 1. Introduction

Regular grammars (RGs) have been widely studied in the theory of computation and intensively applied in natural language processing, compiler construction, software design, parsing and formal verification [1,2,3,4]. Despite their importance and pervasiveness, there is limited research [5,6,7] investigating the internal structure of regular grammars. As such, our understanding of regular grammars is relatively coarse-grained.

One approach to understand a more fine-grained understanding of regular grammars is to investigate them through machine learning. Recent research has demonstrated that recurrent neural networks (RNNs) can achieve superior performance in a wide array of areas that involve sequential data [8], e.g., financial forecasting, language processing, program analysis and particularly grammatical inference. Specially, recent work has shown that certain types of regular grammars can be more easily learned by recurrent networks [9,10]. This is important in that it provides crucial insights in understanding regular grammar complexity. Furthermore, understanding the learning process of regular grammar also help differentiating different recurrent models [11,12,13].

Our intent is to establish a closer connection between the complexity of regular grammars and machine learning tasks from both theoretical and empirical perspectives. From a theoretical perspective, we regard classification and representation as two fundamental problems (Izrail Gelfand once remarked, “all of mathematics is some kind of representation theory” [14]). We follow previous work [15] by studying a grammar from its concentric ring graph representation, which contains sets of strings (with a certain length) accepted and rejected by this grammar. Note that this representation can be used for any grammar. An entropy value is then introduced based on the properties of the concentric ring graph that categorizes all regular grammars into three classes with different levels of complexity, and further establishes several classification theorems based on different representations of regular grammars. In addition, through an empirical study, different regular grammars are categorized by applying them to a set of learning tasks. That is, given enough positive (accepted) and negative (rejected) string samples of a specific regular grammar, it is expected that machine learning models will gradually identify a latent pattern of a grammar through the training process. This shows that this indeed reflects the difficulty of learning regular grammars and highly depends on the complexity of a grammar. All RNNs were evaluated and compared on string sets generated by different RGs with different levels of complexity so as to explore the characteristics of each regular grammar. As such, the empirical results become well aligned with the theoretical analysis. It is hoped that these results can provide a deeper understanding of a regular grammar under a learning scenario as well as practical insights into its complexity.

In summary, the contributions of this paper are as follows:

- We propose a new entropy metric for measuring the complexity of regular grammar in terms of machine learning which reflects the internal structure and hence provides a fine-grained understanding of a regular grammar.
- We categorize regular grammars into three disjoint subclasses based on the entropy value with several classification theorems for different representations proved.
- We empirically demonstrate the validity of the proposed metric and corresponding classification by a series of experiments on the Tomita grammars.

The rest of the paper is organized as follows. Section 2 provides preliminary and background material on recurrent neural networks and regular grammars. Section 3 surveys relevant work on complexity and classification of regular grammars. Section 4 presents our complexity metric and categorization results with several theorems based on different representations proved. Case study and experimental results are shown in Section 5 and Section 6, respectively. Section 7 is the conclusion and future work.

## 2. Preliminaries

First, there is a brief review of fundamental recurrent neural machine learning models. The definition of regular grammars is then introduced, followed by the set of Tomita grammars that are used in this study.

### 2.1. Recurrent Neural Networks

A unified view of the update activity of recurrent neurons for different RNNs investigated is shown in Table 1. Typically, a RNN consists of a hidden layer *h* containing $N_{h}$ recurrent neurons (each designated as $h_{i}$) and an input layer *x* containing $N_{x}$ input neurons (each designated as $x_{k}$). The values of *h* at *t*th and t−1th discrete times are denoted as $h^{t}$ and $h^{t-1}$. Then, the hidden layer is updated by:

(1) $$h^{t+1}=\phi \left(x^{t},h^{t},W\right),$$

where ϕ is the activation function (e.g., Tanh and Relu) and *W* denotes the weights which modify the strength of interaction among input neurons, hidden neurons, output neurons and any other auxiliary units. The hidden layer update for each RNN is presented in Table 1. Next, we describe fundamental RNN models used in our analysis.

#### 2.1.1. Elman Network (SRN)

SRN [16] integrates the input layer and the previous hidden layer in a manner that is regarded as a “first-order” connection [17]. This first-order connection has been widely adopted for building different recurrent layers, for example the gate units in LSTM [18] and GRU [19].

#### 2.1.2. Second-Order RNNs (2-RNN)

2-RNN [20] have second-order connections in their recurrent layers and are designed to capture more complex interactions between neurons. The 2-RNN has a recurrent layer updated by a weighed product of input and hidden neurons. This type of connection enables a direct mapping between 2-RNN and a DFA [21]. Recent work [22] also shows the equivalence between a 2-RNN with linear hidden activation and weighted automata. Related work [23,24] used weighted automata to explore the relationships between deep learning and grammatical inference. Since a 2-RNN has a 3D tensor weight, computation is more intensive. As such, various approximations (M-RNN with a tensor decomposition [25] and MI-RNN with a rank-1 approximation [26]) have been proposed to alleviate the computational cost while preserving the benefits of high order connections for better modeling the complicated recurrent interaction.

#### 2.1.3. RNNs with Gated Units

LSTM [18] and GRU [19] were proposed to deal with the vanishing and exploding gradient problems of SRNs. While these RNNs are effective for capturing the long-term dependence between sequential inputs, their gate units induce highly nonlinear behavior to the update of the hidden layer which creates difficulty in analysis.

### 2.2. Regular Grammar

A regular grammar (RG) recognizes and generates a regular language—a set of strings of symbols from an alphabet—and is uniquely associated with a deterministic finite automata (DFA) with a minimal number of states [27]. For a more thorough and detailed description of regular language and finite state machines, please refer to the work of Hopcroft et al. [27].

The Tomita grammars [28] denote a set of seven regular grammars that have been widely adopted in the study of grammatical inference [29]. Even though the Tomita grammars are relatively simple, they cover regular languages that have a wide range of complexities. and have been widely used as benchmarks [30,31,32] for grammar learning. These grammars all have alphabet Σ={0,1} and generate infinite languages over ${\left\{0,1\right\}}^{*}$. For each Tomita grammar, the binary strings generated by this grammar are its associated positive samples and the rest of the binary strings as negative samples. A description of the positive samples accepted by all Tomita grammars is shown in Table 2. It is important to realize that DFAs cover a wide range of languages, which means that all languages whose string length and alphabet size are bounded can be recognized and generated by a DFA [20]. It is worth noting that regular grammars and finite state machines have many practical uses in mechanical and electrical engineering and software.

## 3. Related Work

Here, we briefly discuss RNNs and regular grammars. We also revisit several complexity measure and some traditional representations of the regular grammar in automata theory.

### 3.1. RNNs and Regular Grammars

Grammars and automata and neural networks have been intertwined since McCulloch and Pitts’ early 1943 paper [33], which led to Kleene’s work [34] on regular grammars. Minsky’s dissertation [35] extended this to models of neural networks and automata. With the rebirth of neural networks, much work on recurrent networks and finite state automata [16,20,36,37] was restarted. Since then, there has been theoretical work on Turing equivalence [38] and finite state machine encoding and representation [39]. The computational hardness of some of the representation results have recently been discussed [12]. Recently, there has been a renewed interest in extracting learned DFA from recurrent neural networks [31,40,41]. Such methods can be important for verification for neural networks and explainable AI [4,42] There has been an increase in the natural language processing community on revisiting formal language models [43]. Because of their wide use and applications, we only focus on regular grammars.

### 3.2. Complexity of Regular Grammars

#### 3.2.1. Complexity of Shift Space

In symbolic dynamics [44], a particular form of entropy is defined to measure the “information capacity” of the *shift space*, which is a set of bi-infinite symbolic sequences that represent the evolution of a discrete system. When applied to measure the complexity of a RG, this entropy describes the cardinality of the strings defined by its language.

#### 3.2.2. Logical Complexity

RG can also be categorized according to logical complexity [45]: strictly local (SL), strictly piecewise (SP), locally testable (LT), etc. These classes have multiple characterizations in terms of logic, automata, regular expressions and abstract algebra [6]. SL and SP languages are the simplest and most commonly used languages that define a finite set of factors and subsequences, respectively, and are selected to evaluate different RNNs on their performance in capturing the long-term dependency [6]. Unlike logical categorizations of regular grammars, we evaluate the complexity in terms of machine learning tasks.

### 3.3. Representations of Regular Grammars

**Figure 1:**
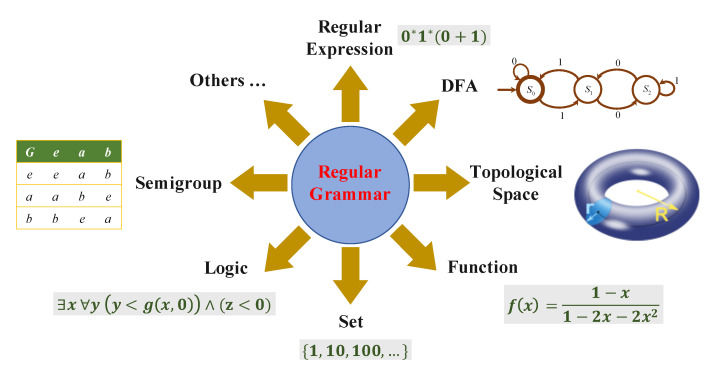
Different representations of regular grammar.

Automata theory has a close connection to many different mathematical subjects such as symbolic dynamics, group theory, algebraic geometry, topology and mathematical logic [46]. Hence, a regular grammar has different representations based on these perspectives. A description of several commonly used representations is shown in Figure 1. We introduce the necessary concepts when they are used in the paper.

### 3.4. Entropy and Regular Grammars

It is important to note that the concept of entropy was introduced in the field of grammatical inference to solve different tasks [47,48] and more recently for distilling weighted automata [49]. Unlike those definitions, our definition originates in the graphical representation of regular grammar and gives insight into a metric for evaluating RNN learning and DFA extraction.

## 4. Categorization of Regular Grammars

**Figure 2:**
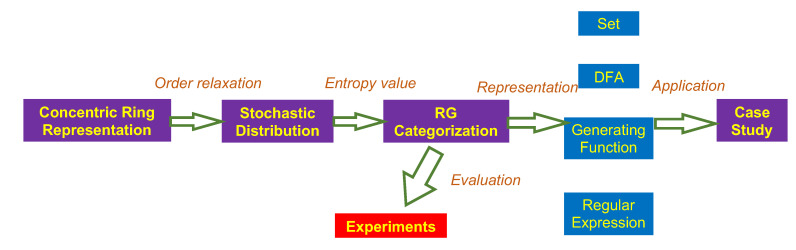
Flowchart of analysis.

This section reviews the concentric ring representation of regular grammar, and, using this representation, we introduce an entropy metric to evaluate their complexity. All RGs are then categorized into three classes according to their entropy values. Last, we provide several classification theorems of RGs in terms of their different representations. A flowchart of our analysis is shown Figure 2.

### 4.1. Entropy of a Regular Language from an Concentric Ring Representation

**Figure 3:**
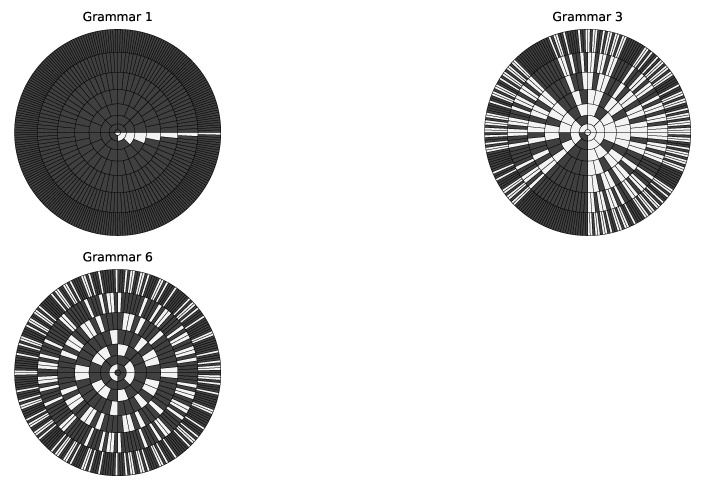
Concentric ring representation of the distribution of strings of length *N* (1≤N≤8) for Grammars 1, 3 and 6. Each concentric ring of a graph has $2^{N}$ strings arranged in lexicographic order, starting at θ=0. (See all graphs in Appendix A.)

The concentric ring representation [15] of a regular grammar reflects the distribution of its associated positive and negative strings within a certain length. Specifically, in each concentric ring, all strings with the same length are arranged in lexicographic order where white and black areas represent accepted and rejected strings respectively. Three ring graphs for Tomita Grammars 1, 3 and 6 are shown in Figure 3 to illustrate their differences. In each graph, every concentric ring contains the sets of strings and its following strings at a specific length that are accepted and rejected by its RG. Note that the percentages of accepted (or rejected) strings for different grammars are very different. For example, Grammars 3 and 6 have the numbers of accepted strings much larger than that of Grammar 1. This difference in prior empirical work [31,41] showed that Grammar 6 is much harder to learn than Grammars 1 and 3. An intuitive explanation is that, for Grammar 6, flipping any 0 to 1 or vice versa means any accepted or rejected string can be converted into a string with the opposite label. A RNN needs to learn such subtle changes in order to correctly recognize all strings accepted by Grammar 6. Since this change can happen to any digit, a RNN must account for all digits without neglecting any.

We now formally show that a RG that generates a more balanced set of accepted and rejected strings has a higher level of complexity and appears more difficult to learn. Given an alphabet Σ={0,1}, the collection of all $2^{N}$ strings of symbols from Σ with length *N* is denoted as $X^{N}$. For a grammar *G*, let $m_{p}^{N}$ ($r_{p}^{N}$) and $m_{n}^{N}$ ($r_{n}^{N}$) be the numbers (ratios) of positive and negative strings, respectively. The constraint that all strings are arranged in a lexicographic order is relaxed, which indicates that all strings in $X^{N}$ are randomly distributed. We then denote the expected times of occurrence for an event $F_{N}$—two consecutive strings having different labels—by $E[F_{N}]$. This gives the following definition of entropy for RGs with a binary alphabet.

**Definition** **1** (Entropy). *Given a grammar G with alphabet Σ={0,1}, its entropy is:*

(2) $$\begin{array}{c} H\left(G\right)=\underset{N\to \infty }{\lim\;\sup}\;H^{N}\left(G\right)=\underset{N\to \infty }{\lim\;\sup}\;\frac{1}{N}\;\log_{2}\;E\left[F_{N}\right], \end{array}$$

*where $H^{N}\left(G\right)$ is the entropy calculated for strings with the length of N. (Here, we use lim sup for certain particular cases, i.e., when N is set to an odd value for Grammar 5.).*

Furthermore, the following proposition to efficiently calculate the entropy by expressing $E[F_{N}]$ explicitly becomes:

**Proposition** **1.** (3) $$H\left(G\right)=1+\underset{N\to \infty }{\lim\;\sup}\;\frac{\log_{2}\left(r_{p}^{N}\cdot r_{n}^{N}\right)}{N}.$$

**Proof of Proposition** **1.** Given any concentric ring (corresponding to the set of strings with a length of *N*) shown in Figure 3, let *R* denote the number of consecutive runs of strings and $R_{p}$ and $R_{n}$ denote the number of consecutive runs of positive strings and negative strings in this concentric ring respectively. Then, we have $E\left[F\right]=E\left[R\right]-1=E\left[R_{p}\right]+E\left[R_{n}\right]-1$. Without loss of generality, we can choose the first position as θ=0 in the concentric ring. Then, we introduce an indicator function *I* by $I_{i}=1$ representing that a run of positive strings starts at the *i*th position and $I_{i}=0$ otherwise. Since $R_{p}=\sum_{i=1}^{2^{N}}I_{i}$, we have

(4) $$\;\begin{array}{c} E\left[R_{p}\right]\;=\;\sum_{i=1}^{2^{N}}E\left[I_{i}\right]\;\;\mathrm{and}\;\;E\left[I_{i}\right]\;=\;\left\{\begin{array}{c} m_{p}/2^{N},\;\;\;\;i=1\\ m_{n}m_{p}/2^{N}\left(2^{N}-1\right),\;i\neq 1. \end{array}\right. \end{array}\;$$

As such, we have

(5) $$\;\begin{array}{c} E\left[R_{p}\right]=\frac{m_{p}\left(1+m_{n}\right)}{2^{N}}\;\;\mathrm{and}\;\;E\left[R_{n}\right]=\frac{m_{n}\left(1+m_{p}\right)}{2^{N}}. \end{array}$$

By substituting E[F] into the entropy definition, we have

(6) $$\begin{array}{c} H\left(G\right)=1+\underset{N\to \infty }{\lim\;\sup}\;\frac{\log_{2}\left(r_{p}^{N}\cdot r_{n}^{N}\right)}{N}. \end{array}$$

□

Thus, the metric entropy is well-defined and lies between 0 and 1. Proposition 1 implies that the entropy of an RG is equal to the entropy of its complement, which confirms our intuition in terms of learning task. Without loss of generality, we assume from now on that for all RGs the set of accepted strings has a smaller cardinality. In addition, we conclude that an RG generating more a balanced string sets has a higher entropy value. As such, we can categorize all RGs with a binary alphabet based on their entropy values.

**Definition** **2** (Subclass of Regular Grammar). *Given any regular grammar G with Σ={0,1}, we have:*

*(a)*  G belongs to **Polynomial** class if H(G)=0;
*(b)*  G belongs to **Exponential** class if H(G)∈(0,1); and
*(c)*  G belongs to **Proportional** class if H(G)=1.

Formally speaking, the metric entropy defines an equivalence relation, denoted as ${∼}_{H}$. A subclass of regular grammar can be considered as the equivalence class by the quotient $\mathrm{RG}/{∼}_{H}$, where RG denotes the sets of all RGs. In this way, we have $\mathrm{RG}/{∼}_{H}=\left\{\left[\mathrm{Po}\right],\left[\mathrm{Ex}\right],\left[\mathrm{Pr}\right]\right\}$, where [Po],[Ex],[Pr] denote polynomial, exponential, and proportional classes, respectively.

When compared to the entropy in shift space, which only considers accepted strings, our Definition 1 considers both the accepted and rejected strings. This is more informative and has other benefits. For example, given a dataset with samples uniformly sampled from an unknown dataset, we can then estimate the complexity of this unknown dataset by calculating the entropy of the available dataset. For a *k*-class classification task with strings of length *N*, let $m_{i}$ denote the number of strings in the *i*th class. Then, we have $E\left[F_{N}\right]=2^{N}-\frac{1}{2^{N}}\cdot \sum_{i=1}^{k}m_{i}^{2}$. We can then generalize Definition 1 to a *k*-class classification case by substituting this into Definition 1. However, this can be challenging for the entropy defined for shift space since it can only be constructed in a one-versus-all manner. In addition, the shift space cannot express all RGs, especially for grammars that lack shift-invariant and closure properties [44].

### 4.2. Set Representation

Here, we conduct a combinatorial analysis. Given a regular grammar *G*, we consider it as a set and explore the property of its cardinality. Namely, we have the following theorem:

**Theorem** **1.** *Given any regular grammar G with Σ={0,1}, we have:*

*(a)*  *G belongs to [Po] if and only if $m_{p}^{N}∼P\left(N\right)$, where P(N) denotes the polynomial function of N;*
*(b)*  *G belongs to [Ex] if and only if $m_{p}^{N}∼\beta \cdot b^{N}$ where b<2 and β>0 and $H\left(G\right)=\log_{2}b$; and*
*(c)*  *G belongs to [Pr] if and only if $m_{p}^{N}∼\alpha \cdot 2^{N}$, where α∈[0,1).*

*Here, ∼ indicates that some negligible terms are omitted when N approaches infinity.*

**Proof of Theorem** **1.** In both Definition 2 and Proposition 1, lim sup is used to cover certain particular cases, for instance when *N* is set to odd value for Grammar 5. In the following proof, without loss of generality, lim is used instead of lim sup for simplicity. According to Proposition 1, for any regular grammar *G*, its entropy H(G)∈[0,1]. It can be checked that the maximum value of H(G) is 1 when $r_{p}^{N}=0.5$. In addition, the minimum value of H(G) is 0 and can be reached when $r_{p}^{N}=0$ or 1. However, $r_{p}^{N}=0$ or 1 are only allowed for grammars that either accept or reject any string, hence are not considered in this theorem. As such, in this case, the value of entropy is taken as the minimum when $r_{p}^{N}=1/2^{N}$ or $1-1/2^{N}$. In the following, we only discuss the former case and the latter can be similarly derived.

For each class of grammars, given that their $m_{p}$ takes the corresponding form shown in Theorem 1, the proof for the sufficient condition is trivial and can be checked by applying *L’Hospital’s Rule*. As such, in the following, we only provide a proof for the necessary condition.

From (4), we have:

(7) $$\begin{array}{cc} H(G) & =\lim_{N\to \infty }\frac{\log_{2}\left(m_{p}\cdot 2^{N}-m_{p}^{2}\right)}{N}-1\\ \; & =\lim_{N\to \infty }\frac{m_{p}^{\prime }\cdot 2^{N}+\ln2\cdot 2^{N}\cdot m_{p}-2m_{p}\cdot m_{p}^{\prime }}{\ln2\cdot (m_{p}\cdot 2^{N}-m_{p}^{2})}-1\\ \; & =\lim_{N\to \infty }\frac{m_{p}^{\prime }\cdot 2^{N}+\ln2\cdot m_{p}^{2}-2m_{p}\cdot m_{p}^{\prime }}{\ln2\cdot m_{p}\cdot \left(2^{N}-m_{p}\right)}, \end{array}$$

where $m_{p}^{\prime }$ denotes the derivative of $m_{p}$ with respect to *N*. It is easy to check that $\lim_{N\to \infty }\frac{m_{p}^{\prime }}{m_{p}}$ exists for regular grammars. Then, we separate the above equation as follows:

(8) $$\begin{array}{c} H\left(G\right)=\lim_{N\to \infty }\frac{m_{p}^{\prime }}{\ln2\cdot m_{p}}+\lim_{N\to \infty }\frac{1-\frac{m_{p}^{\prime }}{\ln2\cdot m_{p}}}{\frac{2^{N}}{m_{p}}-1}. \end{array}$$

It should be noted that the second term in the above equation equals 0. Specifically, assuming that $m_{p}$ has the form of $\alpha \cdot b^{N}$ where b<2 (*b* cannot be larger than 2 for binary alphabet), then the denominator of the second term is infinity. If $m_{p}$ has the form of $\alpha \cdot 2^{N}$, then the numerator tends to zero while the denominator is finite. As such, we have

(9) $$\begin{array}{c} H\left(G\right)=\lim_{N\to \infty }\frac{m_{p}^{\prime }}{\ln2\cdot m_{p}}. \end{array}$$

If H(G)=0, then we have $\lim_{N\to \infty }\frac{m_{p}^{\prime }}{m_{p}}=0$, indicating that the dominant part of $m_{p}$ has a polynomial form of *N* hence $m_{p}∼P\left(N\right)$, where P(N) denotes the polynomial function of *N*.

If H(G)=t≠0, then we have $\lim_{N\to \infty }\frac{\ln(m_{p})}{tN\ln2}=1$, which gives that $m_{p}∼\beta \cdot 2^{tN}$, where β>0. If $t=\log_{2}b$, then we have $m_{p}$$∼\beta \cdot b^{N}$ where b<2. Furthermore, if t=1, we have $m_{p}∼\alpha \cdot 2^{N}$ where α∈[0,1).    □

Theorem 1 shows the reason for naming these subclasses. In addition, when a specific order is posed on the set of given RG, we have a similar result from number theory. Specifically, given a string $s=x_{1}x_{2}x_{3}\cdots x_{N}$ with length *N*, we associate the string with a decimal number, i.e., $D_{s}=\sum_{i=1}^{N}x_{i}\cdot 2^{i-1}+2^{N}-1$. Note that this formula is different from the traditional approach to transforming a binary number to a decimal number, since 0 has physical meaning in regular grammar. For example, 000 is different from 00 in regular grammar. That is the reason that we need an additional term term $2^{N}-1$ to differentiate these situations. In this way, $D_{s}$ has induced an order on the regular grammar. Let $D_{s}^{n}$ denote the *n*th smallest number in this set; then, we have the following result:

**Corollary** **1.** *Given any regular grammar G with Σ={0,1}, we have:*

*(a)*  *G belongs to [Po] if and only if $\lim_{n\to \infty }B_{n}=0$;*
*(b)*  *G belongs to [Ex] if and only if $\lim_{n\to \infty }B_{n}\in \left(0,1\right)$; and*
*(c)*  *G belongs to [Pr] if and only if $\lim_{n\to \infty }B_{n}=1$.*

*Here, $B_{n}=log_{2}n/\log_{2}D_{s}^{n}$.*

Here, we provide a brief explanation of Corollary 1. The denominator approximates the length of the string and the numerator represents the cardinality of the set. Hence, comparing with the original Definition 2, this corollary provide an alternative perceptive of entropy. Take Tomita Grammar 1 for example. The set is G={ϵ,1,11,111,⋯} with the number $D_{s}=\left\{0,2,6,14,\cdots \right\}$, and  the formula for $D_{s}^{n}$ is given by $D_{s}^{n}=2^{n}-2$; hence, by simple calculation, we find that the limit of the ratio is 0 when *n* approaches to infinity, which implies that Grammar 1 belongs to the polynomial class. Note that in general it is difficult to calculate the explicit formula for $D_{s}^{n}$ and therefore Corollary 1 has certain limitation in practical applications.

### 4.3. DFA Representation

Here, we provide an alternative way to obtain the classifications for RGs using the transition matrices of its associated minimal DFA [27] in terms of states. This approach provides immediate results if the minimal DFA is available. As such, this reduces the computation cost of a data-driven approach. Here, we again use the case when the alphabet size is two. However, it is easy to extend this for grammars with larger alphabets. Given a regular grammar *G* with the alphabet Σ={0,1} and its associated minimal complete DFA *M* with *n* states, let $T_{0}$, $T_{1}\in Z^{n\times n}$ denote the transition matrices of *M* associated with input 0 and 1, and  the transition matrix of the DFA is defined as $T=T_{0}+T_{1}$. Alternatively, the transition matrix of a given DFA can be considered as the transition matrix of underlying directed graph by neglecting the labels associated with the edges. With the above settings, we have the following theorem:

**Theorem** **2.** *Given any regular grammar G with Σ={0,1}, we have:*

*(a)*  *G belongs to [Po] if and only if k(T)=1 and σ(T)={1,2};*
*(b)*  *G belongs to [Ex] if and only if k(T)=1 and σ(T)−{1,2}≠∅, and  $H\left(G\right)=\log_{2}\left|\lambda_{2}\right|$ where $\left|\lambda_{2}\right|$ denotes the second largest modulus of the eigenvalues of T; and*
*(c)*  *G belongs to [Pr] if and only if k(T)=0 or k(T)=2.*

*Here, k(T) represents the number of diagonal elements equal to *2* and σ(T) denotes the set of modulus of all eigenvalues of T.*

**Proof of Theorem** **2.** We first introduce a lemma which is used in the proof.

**Lemma** **1.** *Let $v_{1}$ and $v_{2}$ denote two one-hot encoded column vectors corresponding to a starting state and an ending state of a DFA respectively. One can construct an adjacent matrix P for this DFA by regarding it as an undirected graph with each node represents a state and every edge represents the existence of a transition between a pair of states. Then, the number of L-length strings that reach the ending state $v_{2}$ from $v_{1}$ is $v_{1}^{T}P^{N}v_{2}$.*

Based on the lemma above, it is easy to see that the number of positive strings is $m_{p}=v_{1}^{T}P^{N}q$ where *q* is a one-hot encoded column vector representing an accepting state. By applying Jordan decomposition to *P*, i.e., $P=SJS^{-1}$, we can see that $m_{p}=v_{1}^{T}SJ^{N}S^{-1}q$ and $J^{N}$ is the only term depending on *N*. Specifically, take one Jordan block $J_{i}\in R^{m\times m}$ with the eigenvalue of λ and let $K_{i}$ denote the nilpotent matrix associated with $J_{i}$, that is, the superdiagonal of $K_{i}$ contains ones and all other entries are zero. Then, we have:

(10) $$\begin{array}{c} J_{i}{\left(\lambda \right)}^{N}={\left(\lambda I+K_{i}\right)}^{N}=\sum_{n=0}^{\min(N,m-1)}\left(\begin{array}{c} N\\ n \end{array}\right)\lambda^{N-n}K_{i}^{n}. \end{array}$$

It is easy to see that when the absolute of the eigenvalue, i.e., $\left|\lambda \right|=1$, $J_{i}{\left(\lambda \right)}^{N}$ has a polynomial form of *N*. This result can be generalized to all Jordan blocks of *J*. As shown in the proof of Theorem 1, this corresponds to the case when *G* belongs to the polynomial class (we omit the proof since the number of diagonal elements of *T* equal to 2 is 1, as discussed in the paper). Denote the second largest eigenvalue of *T* as *b*, then $\beta \cdot b^{N}$ dominates $m_{p}$, where β is some constant. As such, one can easily derive the proof by following the proof for the exponential class in Theorem 1. □

Theorem 2 indicates that the entropy of a RG lies in the spectrum of its associated DFA. Specifically, in the polynomial and exponential classes, a DFA with its summed transition matrix *T* having only one diagonal element that is equal to 2 indicates that this DFA has only one “absorbing” state (either the accepting state or the rejecting state). Assume that a DFA has one absorbing state and is running over a string. Once reaching the absorbing state, this DFA makes a permanent decision—either acceptance or rejection—on this string, regardless of the ending symbol has been read or not. In comparison, in the proportional class, a DFA can have either zero or two absorbing states (one accepting state and one rejecting state). In the case of Tomita grammars, every grammar has exactly one absorbing state except for Grammars 5 and 6, which have no absorbing states. The DFAs for Grammars 5 and 6 can only determine the label of a string after processing the entire string.

Recall that we require that for all RGs the set of accepted strings has a smaller cardinality. As such, the incomplete DFA for a given RG can be obtained by deleting the rejecting absorbing state and all the edges associated with the state from a minimal complete DFA. (This is important since for a proportional class there might exist two absorbing states and the accepting absorbing state in that case cannot be deleted. A more significant result is that, in the monoid representation of regular grammar, only the monoid associated with polynomial and exponential grammar is a nulloid. The discussion of monoid representation is out scope here.) Similarly, the transition matrix $\hat{T}$ of the incomplete DFA becomes:

**Corollary** **2.** *Given any regular grammar G with Σ={0,1}, we have:*

*(a)*  *G belongs to [Po] if and only if $L(\hat{T})=1$;*
*(b)*  *G belongs to [Ex] if and only if $L\left(\hat{T}\right)\in \left(1,2\right)$ and $H\left(G\right)=\log_{2}\left|L(\hat{T})\right|$; and*
*(c)*  *G belongs to [Pr] if and only if $L(\hat{T})=2$.*

*Here, $L(\hat{T})$ denotes the largest eigenvalue of $\hat{T}$.*

Corollary 2 together with Theorem 2 provides a complete analysis of classification results for a DFA representation. It is easy to see that neither strictly local or strictly piecewise belongs to the proportional class since they all have one absorbing-rejecting state. They can be categorized into either the polynomial or exponential class according to their specific forbidden factors or subsequences. Please refer to classification theorems of regular grammars with arbitrary alphabet size in Appendix B.

### 4.4. Generating Function Representation

Now, we derive the classification results based on the generating function representation of a regular grammar. A generating function encodes an infinite sequence of numbers by treating them as the coefficients of a power series, which is widely applied in combinatorial enumeration. Specifically, the generating function of regular grammar is defined as follows:

(11) $$f\left(x\right)=\sum_{N}m_{p}^{N}\cdot x^{N},$$

An important result is that generating function of regular grammar is a rational function. We have the following theorem for classification:

**Theorem** **3.** *Given any regular grammar G with Σ={0,1}, we have:*

*(a)*  *G belongs to [Po] if and only if $\left\{m_{p}^{N}\right\}$ is finite or the radius of convergence r of f(x) is equal to 1;*
*(b)*  *G belongs to [Ex] if and only if the radius of convergence r of f(x) is between 1/2 and 1, and  $H\left(G\right)=-\log_{2}r$; and*
*(c)*  *G belongs to [Pr] if and only if the radius of convergence r of f(x) is equal to 1/2.*

The theorem can be understood from two perspectives. First, from Theorem 1, we can readily derive the radius of convergence by a ratio test. Another is related to calculating the generating function from a DFA. Specially, we have the following lemma [50]:

**Lemma** **2.** *The generating function $f_{ij}\left(x\right)$ from state i to state j is given by*

(12) $$f_{ij}\left(x\right)=\frac{{\left(-1\right)}^{i+j}\det\left(I-xT:j,i\right)}{\det(I-xT)},$$

*where (B:j,i) denotes the matrix obtained by removing the jth row and ith column of B.*

Note that the radius of convergence *r* only depends on the denominator of the function f(x), and  *r* is the smallest pole of the function. By Lemma 2, the denominator has the form det(I−xT). As such, the radius is the inverse of the largest eigenvalue of the transition matrix. The classification theorem is a generating function of a regular grammar and can be easily generalized to a regular grammar with multiple alphabets.

### 4.5. Regular Expression Representation

Here, we provide an analysis from a regular expression perspective. First, we consider the following interesting question: Given a regular grammar, how many parameters does one need to uniquely determine a positive string? The question is closely related to automatically generate positive samples by computer. We start with a simple example. For Tomita Grammar 7, we have $G=0^{*}1^{*}0^{*}1^{*}$, and  we only need four numbers a,b,c,d to generate a string $0^{a}1^{b}0^{c}1^{d}$. However, for a more complicated example Tomita Grammar 4, we have $G=\left(\epsilon +0+00\right){\left(1+10+100\right)}^{*}$, and, in this case, we have to record both the number and location of the suffix string 1, 10, 100 separately. Hence, we need $Z^{3}$ numbers to generate a sample. Moreover, we have the following fact:

**Fact** **1.** *Given any regular grammar G with Σ={0,1}, we have:*

*(a)*  *G belongs to [Po] if and only if N(G)=k;*
*(b)*  *G belongs to [Ex] if and only if $N\left(G\right)=Z^{k}$; and*
*(c)*  *G belongs to [Pr] if and only if $N\left(G\right)=Z^{Z^{k}}$.*

*Here, k is a constant and N(G) denotes the required number of parameters.*

Another perspective to explore this problem is to apply group theory. Specifically, given a regular grammar, we can consider it as a topological space. Furthermore, it can be decomposed into several disjoint orbits of different base points, and the whole topological space is generated by the actions on these base points. In this way, these actions form a monoid and the cardinality of the monoid reflects the complexity of the grammar. Again, we use Tomita Grammars 4 and 7 as an example to illustrate this idea. For $G=0^{*}1^{*}0^{*}1^{*}$, the base point of the topological space is ϵ and we define the following different actions: $h_{1}$, adding 0 in the first slot; $h_{2}$, adding 1 in the second slot; $h_{3}$, adding 0 in the third slot; and $h_{4}$, adding 1 in the fourth slot. Note that first adding 1 in the second slot and then adding 0 in the first slot is exactly the same as first adding 0 in the first slot and then adding 1 in the second slot, which means that these actions commute to each other. Hence, this monoid is an abelian monoid (more formally, this monoid can be considered as the quotient of a free monoid by a normal submonoid, i.e., $H=F\left\{h_{1},h_{2},h_{3},h_{4}\right\}/<h_{i}h_{j}h_{j}^{-1}h_{i}^{-1}>,\forall i\neq j$) and the cardinality of the monoid is $Z^{4}$. For $G=\left(\epsilon +0+00\right){\left(1+10+100\right)}^{*}$, the base points are ϵ, 0 and 00, and we define the following actions: $h_{1}$, attaching 1 in the back; $h_{2}$, attaching 10 in the back; and $h_{1}$, attaching 100 in the back. Note that this monoid *H* is no longer commutative, since $h_{1}h_{2}$ acting on base point 0 gives the string 0101 while $h_{2}h_{1}$ acting on base point 0 gives the string 0011. Hence, in this case, the monoid is a free monoid generated by $h_{1},h_{2},h_{3}$ and the cardinality of this monoid is $3^{Z}$. More generally, we have the following fact:

**Fact** **2.** *Given any regular grammar G with Σ={0,1}, we have:*

*(a)*  *G belongs to [Po] if and only if C(H)=k or $C\left(H\right)=Z^{k}$;*
*(b)*  *G belongs to [Ex] if and only if $C\left(H\right)=k^{Z}$; and*
*(c)*  *G belongs to [Pr] if and only if $C\left(H\right)=k^{Z^{Z}}$.*

*Here, k is a constant and C(H) denotes the cardinality of the monoid of actions on the topological space.*

Facts 1 and 2 give the following theorem:

**Theorem** **4.** *Given any regular grammar G with Σ={0,1}, we have:*

*(a)*  G belongs to [Po] if and only if there is no + inside the Kleene star * in the regular expression;
*(b)*  G belongs to [Ex] if and only if there exists a polynomial grammar inside the Kleene star * in the regular expression; and
*(c)*  G belongs to [Pr] if and only if there exists an exponential grammar inside the Kleene star * or ${\left(0+1\right)}^{k*}$ in the regular expression, where k is a constant.

Theorem 4 provides an analysis based on the regular expression. Note that, except for the special case in proportional class, this result does not depend on the size of alphabet. More examples are presented in the next section.

## 5. Case Study for Tomita Grammars

As our goal is to study the characteristics of subclasses of regular grammar in terms of machine learning, we use the often cited Tomita grammars [51] as examples for our analysis, which is consistent with the evaluation. For each classification result in Section 4, we choose an example to illustrate its application. The full results for the Tomita grammars are listed in Table 3.

### 5.1. Set Representation

Here, we use Tomita Grammar 3 to illustrate the application of Theorem 1. Note that, in Table 3, we can see that it is difficult to derive an explicit formula for $m_{p}^{N}$ from a pure combinatorial approach. Instead, we provide a brief proof that Grammar 3 belongs to exponential class. Let $G^{N}$ denote the set of positive samples with length N, and it is easy to notice that it can be decomposed into the following four disjoint sets: samples ending with consecutive even numbers of 1 s, samples ending with consecutive odd numbers of 1 s, samples ending with 0 s and the number of 0 s at the end limited by the constraint, and samples ending with 0 s and the number of 0 s at the end not limited by the constraint. We use $A_{1}^{N}$, $A_{2}^{N}$, $A_{3}^{N}$,and $A_{4}^{N}$ to denote these sets, respectively. Hence, we have that $m_{p}^{N}=A_{1}^{N}+A_{2}^{N}+A_{3}^{N}+A_{4}^{N}$. Furthermore, we have the following recursion formulas:

(13) $$\begin{array}{cc} \; & A_{1}^{N}=A_{2}^{N-1},\\ \; & A_{2}^{N}=A_{1}^{N-1}+A_{3}^{N-1}+A_{4}^{N-1},\\ \; & A_{3}^{N}=\sum_{i=1}^{\left[(N-1)/2\right]}A_{2}^{N-2i},\\ \; & A_{4}^{N}=A_{4}^{N-1}+A_{1}^{N-1}, \end{array}$$

where [·] denotes the floor function and the initial condition is $A_{1}^{0}=0$, $A_{2}^{0}=1$, $A_{3}^{0}=0$, and $A_{4}^{0}=1$. By adding these formulas, we have $m_{p}^{N}=m_{p}^{N-1}+m_{p}^{N-2}+A_{2}^{N-2}$, which implies that this sequence increases faster than Fibonacci sequence. Hence, it cannot belong to the polynomial class.

In contrast, let $Z_{1}$ denote the event that an even number of 0 s followed by odd number of 1 s and $Z_{2}$ denotes the event that even number of 0 s always followed by odd number of 1 s in the whole string. Hence, when the length of the string *N* approaches infinity, the expectation of the probability of $Z_{2}$ is an infinite product of probability of $Z_{1}$, which approaches zero. The indicates that the ratio of positive samples approaches zero when *N* approaches infinity. Thus, $m_{p}^{N}$ does not contain the term $\alpha \cdot 2^{N}$. Finally, we conclude that this grammar belongs to exponential class.

### 5.2. DFA Representation

**Figure 4:**
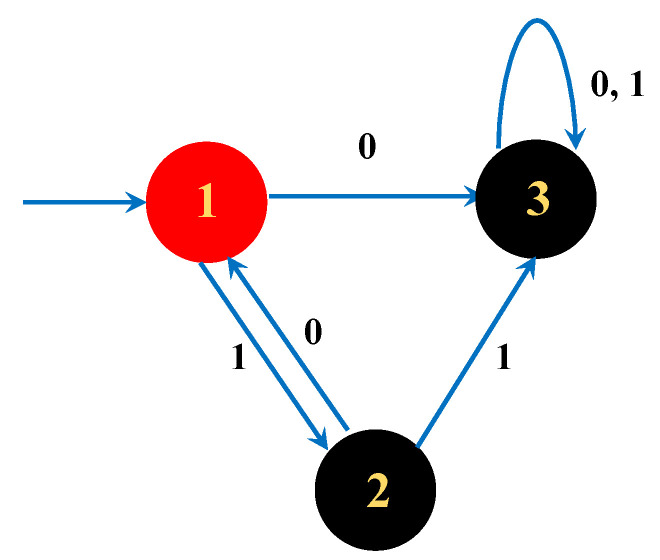
Example for the Tomita Grammar 2 where red (black) states are the accept (reject) states.

Here, we use Tomita Grammar 2 to illustrate the application of Theorem 2. The transition matrix of the complete DFA is $T=\left[\begin{array}{ccc} 0 & 1 & 1\\ 1 & 0 & 1\\ 0 & 0 & 2 \end{array}\right]$ obtained from Figure 4.

By simple calculation, we have k(T)=1 and σ(T)={1,2}. In addition, the transition matrix of the incomplete DFA is $\hat{T}=\left[\begin{array}{cc} 0 & 1\\ 1 & 0 \end{array}\right]$, and  the largest eigenvalue is 1, which means it belongs to the polynomial class.

### 5.3. Generating Function Representation

Here, we use Tomita Grammar 4 to illustrate the application of Theorem 3. The generating function is $f\left(x\right)=\frac{1+x+x^{2}}{1-x-x^{2}-x^{3}}$, and  the radius of convergence is the smallest positive pole of the function. Namely, we need to solve the equation $x^{3}+x^{2}+x-1=0$ and we have r=0.544, which lies between the values of 0 and 1. Hence, it belongs to the exponential class.

### 5.4. Regular Expression Representation

Here, we use Tomita Grammar 5 to illustrate the application of Theorem 4. For $G={\left(00+11\right)}^{*}{\left(\left(01+10\right){\left(00+11\right)}^{*}\left(01+10\right){\left(00+11\right)}^{*}\right)}^{*}$, there exists an expression $G^{\prime }=\left(01+10\right){\left(00+11\right)}^{*}\left(01+10\right){\left(00+11\right)}^{*}$ inside the Kleene star. For grammar $G^{\prime }$, there exists a polynomial grammar 00+11 inside the Kleene star, indicating that $G^{\prime }$ is an exponential grammar. Hence, *G* belongs to the proportional class.

## 6. Evaluation

Here, our empirical results show that the categorization of RGs is related to the difficulty of RNNs to learn these grammars, and the implementation is publicly available (https://github.com/lazywatch/rnn_theano_tomita.git). We evaluated the three Tomita Grammars 1, 3 and 5. For each grammar, its subclass is shown in Table 3. Following prior work [31], we generated three training sets of binary strings with their lengths ranging from 1 to 14 for these grammars. We also collected three validation sets of strings with different lengths of [1, 4, 7, ⋯, 25], to make sure that the models can be trained to generalize to longer strings. The training process was terminated either when the model achieved a $F_{1}$-score that is equal or higher than 0.95 or when a maximum of 5000 epochs were reached. We selected several different recurrent networks to demonstrate how the difficulty of learning generalizes to different models. Specifically, we trained SRN, 2-RNN, GRU and LSTM with data generated on each grammar to a perform a binary classification task. We configured all RNNs to have the same size of hidden layer across all grammars and trained them on each grammar for 10 random trials using a mean squared error loss. In each trial, we randomly initialized the hidden layer of the model.

We followed previous research [20] and used either activation functions—sigmoid and tanh—to build these RNNs. In addition, for each RNN, we used one-hot encoding to process the input alphabets of 0 s and 1 s. With this configuration, the input layer is designed to contain a single input neuron for each symbol in the alphabet of the target language. Thus, only one input neuron is activated at each time step. Moreover, we followed the approach introduced in previous research and applied the following loss function to all RNNs:

(14) $$\begin{array}{c} L=\frac{1}{2}{\left(y-h_{0}^{T}\right)}^{2}. \end{array}$$

This loss function can be viewed as selecting a special “response” neuron $h_{0}$ and comparing it to the label *y*, i.e., 1 for acceptance and 0 for rejection. Thus, $h_{0}^{T}$ indicates the value of the neuron $h_{0}$ at time *T* after a RNN receives the final input symbol. By using this simple loss function, we attempt to eliminate the potential effect of adding an extra output layer and introducing more weight and bias parameters. In addition, by this design, we ensure that the knowledge learned by a RNN resides in the hidden layer and its transitions. When applying different activation functions, we make sure that $h_{0}$ is always normalized between the range of 0 and 1, while other neurons have their values between 0 and 1 for sigmoid activation and −1 to 1 for tanh activation. During training, we optimized parameters through stochastic gradient descent and employed RMSprop optimization [52].

**Figure 5:**
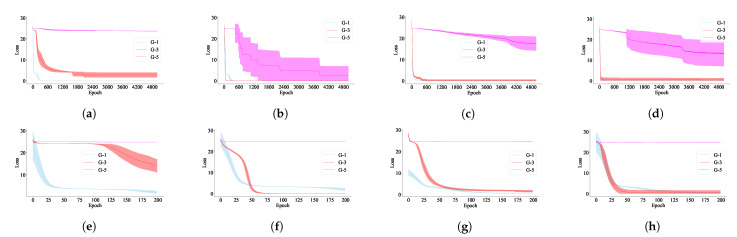
Error loss vs. epochs on Tomita Grammars 1, 3 and 5. (**a**) SRN. (**b**) 2-RNN. (**c**) LSTM. (**d**) GRU. (**e**) SRN-zoom (**f**) 2-RNN-zoom. (**g**) LSTM-zoom. (**h**) GRU-zoom.

The results are shown in Figure 5, where the results from all trials fit the shaded area associated with each plot. The *x*-axis represents the number of epochs during training and the *y*-axis represents the loss. In Figure 5a–d, we see that Grammars 1 and 3, which have lower entropy values, have learning that converges much more quickly and consistently than that of Grammar 5, which has the highest entropy value. This effect holds for all RNNs evaluated. Note that Grammar 5 defines two sets of strings with equal cardinality when the string length is even. In this case, by flipping any binary digit of a string to its opposite (e.g., flipping a 0 to 1 or vice versa), a valid or invalid string can be converted into a string with the opposite label. This implies that a model must pay equal attention to any string in order to learn the underlying grammar, which makes the learning process more challenging.

To better illustrate the difference of the difficulty of learning Grammars 1 and 3, a zoomed view is provided in Figure 5e–h, for each plot at the top row of Figure 5. While the learning process of all models converges within 100 epochs for both grammars, it is clear that the learning process is slower for Grammar 3. These results agree with both our analysis of the entropy of these grammars and our intuition.

It would be interesting to further investigate the relationship between recurrent models and grammar learning, which is out of the scope for this paper. A promising approach would be to investigate their connection is to more closely represent DFA representations, since both are stateful models. In general, while it is possible to validate the connection between nonlinear RNNs and DFAs empirically, it has been challenging to establish a theoretical connection between nonlinear RNNs and finite state machines. Specifically, second-order RNNs naturally fit into the task of learning any DFA, while some first-order RNNs only represent a portion of DFAs, indicating that the gap (not computationally) between first-/second-order RNNs is not as significant as expected. However, for these gated models (LSTM/GRU), we only observe the differences by experiments.

From the evaluation results, a more general question is to establish an analysis between different types of RNNs and DFAs. To better study this, we discuss the following two questions: Given a RG, does there exist some common substrings misclassified by different RNNs? Given a RNN, are there consistent persistent flaws when learning different RGs? Our results observed on the Tomita grammars can be considered as an initial attempt to answer the above questions. We first find that all RNNs perform perfectly on polynomial RGs (e.g., Grammars 1, 2 and 7), and first-order RNNs perform poorly on proportional RGs (e.g., Grammars 5 and 6). In addition, for first-order RNNs, both their overall classification performance and misclassified strings indicate that these RNNs randomly choose some RGs to learn when learning proportional RGs. For exponential RGs (e.g., Grammar 3), we find that there exist some patterns in strings misclassified by certain first-order and gated RNNs. For example, LSTM tends to ignore “101” appearing at the end of a string and subsequently a has high false-positive errors (and learns some less deterministic transitions). In contrast, the SRN tends to lose count of consecutive 0 s or 1 s. These results indicate that these RNNs learn a smoothed boundary of the manifold that holds these strings. Since these common substrings are more likely to lie in the periphery of the manifold, it suggests the use of up-sampling to compensate for this problem.

## 7. Conclusions and Future Work

A theoretical analysis and empirical validation for subclasses of regular grammars is presented. Specifically, to measure the complexity of regular grammar, we introduced an entropy metric based on the concentric ring representation, which essentially reflects the difficulty in training RNNs to learn the grammar. Using entropy, we categorized regular grammar into three disjoint subclasses: polynomial class, exponential class and proportional class. In addition, we provided classification theorems for different representations of regular grammar. Given a regular grammar, these theorems use its corresponding properties in the given representation to efficiently determine which subclass a grammar belongs to without calculating the entropy value. Several common representations including deterministic finite automata, regular expression and sets have a corresponding case study which illustrates their applications. This categorization could also be applied to other relevant representations. Finally, we conducted an experiment to demonstrate the influence of grammar learning based on its complexity, which validates the effectiveness of the proposed entropy metric and the theoretical analysis. All RNNs have problems learning certain classes of grammars. It would seem the grammar chosen matters more than the RNN architecture. We believe this work provides a deeper understanding of the internal structure of regular grammar in the context of learning.

Future work could include an extension to other types of grammars, e.g., context-free grammars. The concentric ring representation is independent of grammar type and can be similarly applied to context-free grammars giving similar results to their entropy. However, classification differs dramatically. For instance, when we consider the parentheses grammar, the entropy can be obtained by the central binomial coefficient, which fails to fall in any of the classes proposed in this work. Another perspective is to study grammar learning in terms of recurrent models. Such a connection between DFA and RNNs can provide insights into explainable artificial intelligence and adversarial machine learning.

## Figures and Tables

**Table 1:** Hidden update of RNNs selected; building blocks for developing many complicated models. Let $W_{*}$, $U_{*}$ and $V_{*}$ denote weights designed for connecting different neurons and *b* denote the bias. ⊙ is the Hadamard product.

Model	Hidden Layer Update ($U_{*}\in R^{N_{h}\times N_{x}},V_{*}\in R^{N_{h}\times N_{h}},b\in R^{N_{h}\times 1}$)
SRN	$h^{t}=\phi \left(Ux^{t}+Vh^{t-1}+b\right)$
2-RNN	$h_{i}^{t}=\phi \left(\sum_{j,k}W_{kij}h_{j}^{t-1}x_{k}^{t}+b_{i}\right),\;i,j=1,\cdots ,N_{h},\;k=1,\cdots ,N_{x}$, $W\in R^{N_{h}\times N_{h}\times N_{x}}$
LSTM	$s^{t}=\phi \left(U_{s}x^{t}+V_{s}h^{t-1}\right)$, s={i,f,o,g} and ϕ={Sigmoid,Tanh}
	$c^{t}=c^{t-1}⊙f^{t}+g^{t}⊙i^{t}$, $h^{t}=\mathrm{Tanh}\left(c_{t}\right)⊙o^{t}$
GRU	$z^{t}=\sigma \left(U_{z}x^{t}+V_{z}h^{t-1}\right)$, $r^{t}=\sigma \left(U_{r}x^{t}+V_{r}h^{t-1}\right)$,
	$g^{t}=\mathrm{Tanh}\left(U_{h}x^{t}+V_{h}\left(h^{t-1}⊙r^{t}\right)\right)$, $h^{t}=\left(1-z^{t}\right)⊙g^{t}+z^{t}⊙h^{t-1}$

**Table 2:** Descriptions of the Tomita grammars. The Kleene star is represented by *.

G	Description
1	1 *
2	(10)*
3	an odd number of consecutive 1 s are always followed by an even number of consecutive 0 s
4	any string not containing “000” as a substring
5	even number of 0 s and even number of 1 s
6	the difference between the number of 0 s and the number of 1 s is a multiple of 3
7	0${}^{*}$$1^{*}0^{*}1^{*}$

**Table 3:** Analysis of Tomita grammars. ($\beta =1/3\cdot {\left(19+3\sqrt{33}\right)}^{1/3}+1/3\cdot {\left(19-3\sqrt{33}\right)}^{1/3}+1/3$ and $b=\left\{3{\left(586+102\sqrt{33}\right)}^{1/3}\right\}/\left\{{\left(586+102\sqrt{33}\right)}^{2/3}+4-2{\left(586+102\sqrt{33}\right)}^{1/3}\right\}$. This is also known as a *Tribonacci number*.)

*G*	$m_{p}^{N}$	$L(\hat{T})$	f(x)	Regular Expression	Class
1	1	1	$\frac{1}{1-x}$	$1^{*}$	Po
2	$0.5+0.5{\left(-1\right)}^{N}$	1	$\frac{1}{1-x^{2}}$	${\left(10\right)}^{*}$	Po
3	-	1.77	$\frac{{\left(1+x\right)}^{2}}{1-2x^{2}-2x^{3}}$	$0^{*}{\left({\left(11\right)}^{+}0^{*}+1{\left(11\right)}^{*}{\left(00\right)}^{+}\right)}^{*}\left(\epsilon +1{\left(11\right)}^{*}\right)$	Ex
4	$\beta \cdot b^{N}$	1.84	$\frac{1+x+x^{2}}{1-x-x^{2}-x^{3}}$	$\left(\epsilon +0+00\right){\left(1+10+100\right)}^{*}$	Ex
5	$2^{N-2}\left(1+{\left(-1\right)}^{N}\right)$	2	$\frac{1-2x^{2}}{1-4x^{2}}$	${\left(00+11\right)}^{*}{(\left(01+10\right)\left(00+11\right)}^{*}$ $\left(01+10\right){\left(00+11\right)}^{*}{)}^{*}$	Pr
6	$(2^{N}-2{\left(-1\right)}^{N})/3$	2	$\frac{1-x}{\left(1-2x)(1+x\right)}$	${\left(10+01\right)}^{*}{(\left(1+00\right)\left(01+10\right)}^{*}$ $\left(11+0\right){\left(01+10\right)}^{*}+\left(11+0\right){\left(01+10\right)}^{*}$ $\left(1+00\right){\left(01+10\right)}^{*}{)}^{*}$	Pr
7	$(N^{3}+5N+6)/6$	1	$\frac{1-2x+2x^{2}}{{\left(1-x\right)}^{4}}$	$0^{*}1^{*}0^{*}1^{*}$	Po

**Figure A1:**
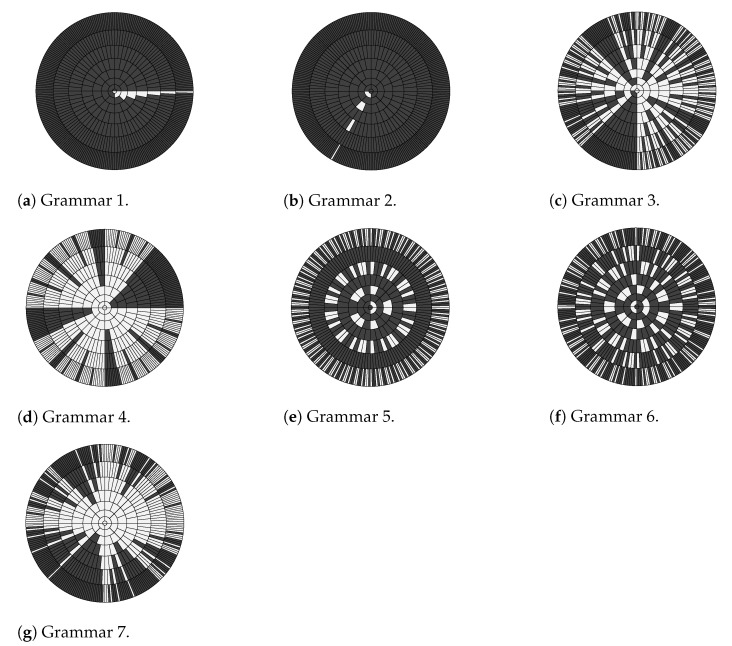
Concentric ring representation of the distribution of strings of length *N* (1 ≤ *N* ≤ 8) for Tomita Grammars. Each concentric ring of a graph has 2^*N*^ strings arranged in lexicographic order, starting at *θ* = 0.

## Data Availability

The implementation is publicly available at https://github.com/lazywatch/rnn_theano_tomita.git.

## Appendix A. Concentric Ring Representation Representation of Tomita Grammars

Here, we plot all graphs for Tomita grammars to illustrate their differences.

## Appendix B. Generalization to the Regular Grammars with an Arbitrary Alphabet

Given a regular grammar *G* with a *I*-size alphabet $\Sigma_{I}$ and its associated minimal DFA *M* with *n* states, let $T_{i}\in Z^{n\times n}$ denote the transition matrix of *M* associated with *i*th input symbol for i∈{1,⋯,I}, and $T=\sum_{i}^{I}T_{i}$ is the sum of all transition matrices. We use k(T) to represent the number of diagonal elements equal to *I* and σ(T) to denote the set of modulus of all eigenvalues of *T*. $\left|\lambda_{2}\right|$ is used to represent the second largest modulus of the eigenvalues of *T*. Then, its categorization result for the three classes introduced in Theorems 1 and 2 is shown in the following table (without loss of generality here we only derive the result using the number of positive samples, i.e., $m_{p}$, defined by this grammar).

**Table A1:** Generalization to the Regular Grammars with an Arbitrary Alphabet.

Category	Data-Driven Perspective	DFA Perspective	Entropy
[Po]	$m_{p}∼P\left(N\right)$ (polynomial of *N*)	k(T)=1 and σ(T)={1,I}	H(G)=0
[Ex]	$m_{p}∼\beta \cdot b^{N}$ where b<I and β>0	k(T)=1 and σ(T)−{1,I}≠∅	$H\left(G\right)=\log_{I}\left|\lambda_{2}\right|\in \left(0,1\right)$
[Pr]	$m_{p}∼\alpha \cdot I^{N}$, where α∈(0,1)	k(T)=0 or k(T)=2	H(G)=1
